# Mitochondrial Dynamics Protein Drp1 Is Overexpressed in Oncocytic Thyroid Tumors and Regulates Cancer Cell Migration

**DOI:** 10.1371/journal.pone.0122308

**Published:** 2015-03-30

**Authors:** André Ferreira-da-Silva, Cristina Valacca, Elisabete Rios, Helena Pópulo, Paula Soares, Manuel Sobrinho-Simões, Luca Scorrano, Valdemar Máximo, Silvia Campello

**Affiliations:** 1 Institute of Molecular Pathology and Immunology of the University of Porto (IPATIMUP), Porto, Portugal; 2 Department of Pathology, Medical Faculty of University of Porto, Porto, Portugal; 3 Department of Biology, University of Rome Tor Vergata, Rome, Italy; 4 Department of Pathology, Hospital S. João, Porto, Portugal; 5 Department of Biology, University of Padua, Padua, Italy; 6 Dulbecco Telethon Institute, Venetian Institute of Molecular Medicine, Padua, Italy; 7 IRCCS Fondazione Santa Lucia, Rome, Italy; Boston University School of Medicine, UNITED STATES

## Abstract

Oncocytic cell tumors are characterized by the accumulation of morphologically abnormal mitochondria in their cells, suggesting a role for abnormal mitochondrial biogenesis in oncocytic cell transformation. Little is known about the reason for the dysmorphology of accumulated mitochondria. The proteins regulating the morphology of mitochondria, the "mitochondria-shaping" proteins, can modulate their size and number; however, nothing is known hitherto about a possible involvement of mitochondrial dynamics in oncocytic cell transformation in tumors. Our aim was to assess the status of the mitochondria morphology and its role in oncocytic cell transformation. We therefore evaluated the expression pattern of the main mitochondrial fusion and fission proteins in a series of thyroid cell tumor samples, as well as in thyroid tumor cell lines, with and without oncocytic cell features. The expression of mitochondrial fusion (Opa1, Mfn1 and Mfn2) and fission (Drp1 and Fis1) proteins were evaluated by immunohistochemistry (IHC) in a series of 88 human thyroid tumors. *In vitro* studies, for comparative purposes and to deepen the study, were performed using TPC1 - a papillary thyroid carcinoma derived cell line—and XTC.UC1, an oncocytic follicular thyroid carcinoma-derived cell line. Both IHC and *in vitro* protein analyses showed an overall increase in the levels of "mitochondrial-shaping" proteins in oncocytic thyroid tumors. Furthermore, overexpression of the pro-fission protein Drp1 was found to be associated with malignant oncocytic thyroid tumors. Interestingly, genetic and pharmacological blockage of Drp1 activity was able to influence thyroid cancer cells’ migration/invasion ability, a feature of tumor malignancy. In this study we show that unbalanced mitochondrial dynamics characterize the malignant features of thyroid oncocytic cell tumors, and participate in the acquisition of the migrating phenotype.

## Introduction

Cancer cells are known to undergo a shift in their basal metabolic pathways, a process often described as the “*Warburg effect*”, whereby even under high oxygen tension they produce most of their ATP by glycolysis[1]. This shift in metabolism has been reported to be accompanied by a change in mitochondrial morphology and size, although the molecular details accompanying the morphological changes associated with the Warburg effect remain unexplained, also since little is known on how mitochondrial morphology is regulated[2,3]. Recent years have seen the identification of the core components of the mitochondrial dynamics machinery, a growing group of proteins which, among other cellular functions, regulate mitochondrial fusion and fission. Mitochondrial dynamics dysfunction has been progressively unveiled in a wide number of pathologies, especially neurodegenerative diseases and cancer[4].

A particular group of uncommon tumors has been reported in virtually every organ, with a higher frequency in thyroid and kidney[5,6]. Such tumors have cells with a distinct highly eosinophilic, grainy and swollen cytoplasm, with large nuclei and prominent nucleoli [7,8,9,10]. Electron microscopy has shown that this swollen appearance is due to an anomalous accumulation of mitochondria displaying abnormal morphology[11,12,13]. These tumors are described as oncocytic-, oxyphilic-, eosinophilic, “Hürthle-cell (when referring to the thyroid) tumors” or as “oncocytomas”. For the sake of simplicity, we will designate them as “oncocytomas” or “oncocytic cell tumors”[10]. In current clinical practice, the criteria for diagnosis of the oncocytic cell variants of papillary thyroid carcinoma (PTC) and follicular thyroid carcinoma (FTC) include nuclear characteristics, signs of capsular and/or vascular invasion which are shared with the non-oncocytic cell tumors of the same kind, as well as the mitochondrial proliferation typical for oncocytic cell tumors[5,6,7]. Indeed, this idea that the oncocytic transformation occurs on top of “conventional” tumors is backed up by the similar genetic alterations of oncocytic carcinomas and their non-oncocytic counterparts, which display a comparable incidence of the various genetic abnormalities, such as RET/PTC rearrangements and BRAFV600E mutations[6,14,15,16,17,18]. Although the first recommendations considered all oncocytic thyroid tumors malignant, later studies have demonstrated that as long as the cases were correctly stratified according to their clinico-pathological features-such as patients’ age, staging of the tumors and surgical procedure- the prognosis of patients with oncocytic cell PTC and FTC variants is similar to that of patients with respective conventional, non-oncocytic carcinomas[3,5,17,19].

The main differences between oncocytic and non-oncocytic tumors reside in the frequency of variations found in mitochondrial DNA (mtDNA) and nuclear DNA genes encoding mitochondrial proteins[2,5,20,21]. Of these, a large 5kb deletion, often referred to as the "common deletion" that eliminates a number of mtDNA genes and disrupts mtDNA replication and transcription, is frequently found in oncocytic thyroid tumors and is associated with an increase in mitochondrial mass[20,22,23,24].

The data on record suggest that mutations in oxidative phosphorylation (OXPHOS) genes, namely in Complex I genes, may result in OXPHOS system impairment and may lead to the accumulation of abnormal mitochondria[20,25]. Using bioinformatic tools, we have recently demonstrated that high pathogenicity mtDNA mutations, namely in genes for Complex I, lead to the oncocytic phenotype[21].

Variants of the Translocase of Inner Mitochondrial Membrane 44 homolog (TIMM44) that is part of the mitochondrial protein import system have also been described in familial forms of oncocytic thyroid tumors; this suggests that deregulation of the mitochondrial import machinery and consequently of the mitochondrial function are associated with abnormal mitochondrial biogenesis[26].

A striking feature of the accumulated mitochondria in oncocytic cell tumors is their abnormal ultrastructure and morphology. This indicates a deregulation also in the proteins that control mitochondrial dynamics, the "mitochondria-shaping" proteins, a process dependent on mitochondrial fusion and fission and inextricably linked to mitochondrial biogenesis, distribution, signaling and apoptosis[27,28,29]. Interestingly, imbalance of the mitochondria phenotype toward fragmentation orchestrates the migratory capability in cells where migration represents a crucial physiological function, such as T-cell lymphocytes and tumoral metastatic cells[30,31]. Indeed, in order to make movement possible, mitochondria need to be fragmented to favor their relocalization and recruitment to specific subcellular regions. In the case of tumoral cells, a direct correlation has been demonstrated between the metastatic degree and the Drp1-dependent fragmentation of mitochondria in breast cancer[31]. Mitochondrial fission requires the cytosolic dynamin related protein 1 (Drp1) and its mitochondrial receptors fission 1 (Fis1), mitochondrial fission factor (Mff) and Mitochondrial Division (Mid) 49 and 51. Conversely, the outer membrane mitofusin (Mfn) 1 and 2 and the inner membrane dynamin-like protein optic atrophy 1 (Opa1), mediate mitochondrial fusion[32,33]. Despite the known mitochondrial morphological alterations observed in oncocytic cell tumors, knowledge about the levels and the role of these mitochondria-shaping proteins is scanty. We therefore set out to investigate if mitochondrial dynamics is altered in oncocytic thyroid cell tumors. Our data indicate that mitochondrial fission proteins Drp1 and Fis1 are overexpressed in oncocytic cell tumours, and that Drp1 expression levels correlates with oncocytic cell tumor malignancy *ex vivo* and with the migration ability of an oncocytic thyroid tumor cell line *in vitro*. Interestingly, genetic and pharmacological blockage of Drp1 reduces the migration of the oncocytic tumor cell line, offering a novel therapeutic approach to tackle metastatic oncocytic cell tumors.

## Materials and Methods

### Ethics Statement

The present study was performed under the national ethical and regulative law for the use of biological specimens. The material used in this study comes from the “Banco de Tecidos e Tumores “(Tumors and Tissues Bank) of the Hospital de São João, Porto, which collects all samples upon written informed consent of the patients, or their guardians in case of the minors. To have access to these samples, researchers must submit a project to the Ethics Committee of the Hospital de São João for approval. Our project has been approved.

### Patient selection

A series of 88 thyroid tumors were retrieved from the files of Department of Pathology of Hospital S. João (HSJ), Porto. Thyroid samples were obtained from patients with ages ranging from 15 to 83 years of age. The histology of all tumor samples was revised independently by two pathologists (ER and MSS) and the final classification was made according to the WHO criteria[34]. The diagnosis of the thyroid tumors were the following: follicular thyroid adenoma (FA, n = 19; 12 oncocytic and 7 non-oncocytic), follicular thyroid carcinoma (FTC, n = 7; 1 oncocytic and 6 non-oncocytic), follicular variant of papillary thyroid carcinoma (FVPTC, n = 30; 8 oncocytic and 22 non-oncocytic), conventional papillary thyroid carcinoma (cPTC, n = 32; 9 oncocytic and 23 non-oncocytic).

Due to the short follow-up of the cases included in this series and to the consequent limited number of death events, overall survival was not analyzed. The present study was performed under the national ethical and regulative law for the use of biological specimens.

### Tissue Microarray (TMA)

Representative areas of different lesions were carefully selected on haematoxylin and eosin-stained sections and were marked on individual paraffin blocks. Two tissue cores (3 mm in diameter each) were obtained from all the selected specimens and were precisely deposited into duplicate TMA recipient paraffin blocks using a tissue-array instrument (Beecher Instruments, Silver Spring, MD) as described elsewhere[35]. In each tissue microarray block, areas of non-neoplastic thyroid tissue were also included as controls. Multiple 3 μm sections were cut from the TMA blocks and transferred to ultrafrost slides.

### Immunohistochemical analysis

Immunohistochemical analysis (IHC) was performed in 3μm formalin-fixed, paraffin-embedded sections. Sections were deparaffinized and rehydrated in a series of decreasing concentration of ethanol solutions. Deparaffinized single specimens as well as TMA sections were subject to heat-induced antigen retrieval either in 10mM pH6 citrate buffer (sodium citrate) or in 1mM pH8 ethylenediamine tetraacetic acid buffer (EDTA) (LabVision Corporation, Fremont, CA, USA), in a microwave set at 300watts for 10 minutes.

After retrieval solutions had cooled, tissues were subject for 10 minutes to blocking of endogenous peroxidase activity and of non-specific binding with 3% of H_2_O_2_ and with the Large Volume Ultra V Block reagent (Thermo Scientific/Lab Vision, Fremont, USA) depending on the detection system used.

The sections were then incubated in a humidified chamber with the following primary antibodies and accordingly with manufacturer’s specifications: rabbit polyclonal antibody specific for Fis1 (1:100) ref. ALX-210-907 from Alexis Biochemicals, now Enzo Life Sciences (Farmingdale, New York, USA), mouse monoclonal antibody specific for Drp1 (1:100) ref. 611112 and mouse monoclonal antibody specific for Opa1 (1:100) ref. 612607, both from BD Biosciences (Franklin Lakes, New Jersey, USA), rabbit polyclonal specific for Mfn1 (1:250) ref. sc-50330 (Santa Cruz Biotechnology Santa Cruz, USA), mouse monoclonal antibody specific for Mfn2 (1:400) ref. 9927-M03 from Abnova, (Jhongli City, Taiwan). As a control for mitochondrial content mouse polyclonal antibody specific for SDHA (1:2000) ref. MS203, from Mitosciences, now abcam (Cambridge, UK) was used. Previously tested positive controls from thyroid, breast and muscle were included for all the antibodies in the series. Negative controls were carried out by replacing the primary antibody with nonimmune mouse serum.

The immunohistochemistry technique was performed using a labeled streptavidin-biotin immunoperoxidase detection system (Thermo Scientific/Lab Vision, Fremont, USA) or the Envision G/2 System/AP (K5355, Dako, Denmark) according to the manufacturer's instructions.

For MFN1 and SDHA the immunohistochemical staining was developed with DAB (3,3'-diaminobenzidine) substrate. For the remaining antibodies the immunostaining was performed with or the Envision G/2 System/AP (K5355, Dako, Denmark), and the samples were developed with a permanent red chromogen.

Immunostaining was blindly semi-quantitatively evaluated by two observers (VM and AFS) without knowledge of any clinical information of the cases; an IHC score was obtained through the sum of intensity of staining (0- absent; 1- faint; 2- moderate; 3- strong) by the extension of stained tumor cells (1–0 to 25%; 2–25 to 50%; 3–50 to 75%; 4- >75%). Evaluation of the percentage of immunoreactive cells for all the antibodies was made by counting 300 tumor cells in random fields. In all discrepant cases a consensus was reached. As mitochondrial marker, and as a mean to assess the amount of mitochondria, SDHA expression levels were accessed and used to normalize our results. In every sample, we compared the expression of the proteins of interest, both in normal and tumor tissue, against SDHA staining. Thus, for each case, we were able to evaluate in an individual and precise manner the changes in expression of the protein in the study independently of the amount of mitochondria, indicated by the SDHA expression.

### Cell lines

Thyroid cancer cell lines: TPC1 (kindly provided by Dumont JE and Mareel M—National Cancer Center Research Institute, Tokyo, Japan), a PTC-derived cell line, and XTC.UC1 (from Wong MG and Savagner F—Surgery Service, Veterans Affairs Medical Center, San Francisco, California), a cell line obtained from an oncocytic variant of follicular carcinoma, were used in this study. Both cell lines had been previously characterized at the molecular and genotypic level[36,37]. TPC1 cells were cultured with RPMI medium with Glutamax supplemented with 10% (v/v) fetal bovine serum (FBS), 1% (v/v) Pen Strep and 0.5% (v/v) fungizone (all from GIBCO, Invitrogen, Carlsbad, USA); XTC.UC1 was maintained with DMEM/F12 (GIBCO, Invitrogen, Carlsbad, USA) supplemented with 10% (v/v) FBS, insulin at 10μg/mL, TSH at 10mU/mL (Sigma-Aldrich, St. Louis, USA), 1% Pen Strep (v/v) and 0.5% (v/v) fungizone. Both cell lines were authenticated using DNA profile analysis, obtained with the PowerPlex 16 system (Promega, Madison, USA), according to the DNA profiles available in American Type Culture Collection and Health Science Research Resource Bank.

### Constructs and transfections

Cytoplasmatic GFP (ctGFP), mitochondrially targeted dsRED (mtRFP), mitochondrially targeted YFP (mtYFP) and pcDNA3.1-HA-K38A-DRP1 plasmids were previously described and were a gift from T. Pozzan (Venetian Institute of Molecular Medicine, Padua, Italy)[33]. The empty pcDNA3.1 was obtained from BD-Clontech and pcDNA3.1-HA-K38A-DRP1 plasmid generation was previously described[33]. XTC.UC1 cell lines were transiently transfected by electroporation using the Neon Transfection System (Life Technologies, Carlsbad, CA, USA). In co-transfections experiments, 1.5μg of marker carrier (ctGFP in transwell experiments) plus 3μg of pcDNA 3.1 or pcDNA3.1-HA-K38A-DRP1 were used per 2.0x10^6^ cells. The specific combination of plasmids transfected in each experiment is indicated in the figure legends. After 24h, transfected cells were sorted by FACS and used for experiments 24h later.

### Quantitative PCR

For cDNA preparation, 1ng of total RNA was reverse transcribed using the RevertAid first strand cDNA synthesis kit (Fermentas, Burlington, ON, Canada). Reverse transcription products were amplified for all the aforementioned genes by qPCR using TaqMan PCR Master Mix (Applied Biosystems, Foster City, CA, USA). For data analysis *Actin* and *hCyclophilin* were used as endogenous controls (data shown only for actin). Data are expressed as relative expression for each individual gene normalized to the corresponding controls.

The ABI PRISM 7500 Fast Sequence Detection System (Applied Biosystems) was used to detect the amplification level and was programmed to an initial step of 2 min at 50°C, 10 min at 95°C, followed by 45 cycles of 95°C for 15 sec and 60°C for 1 min. 50ng of cDNA for each sample was used and all the amplifications were done in triplicate. The relative quantification of target genes was determined using the ∆∆CT method, which was previously validated by Livak’s Linear Regression Method (slope¼0.0696) (Sequence Detector User Bulletin 2; Applied Biosystems).

### Subcellular fractionation and immunoblotting

Subcellular fractionation was performed as described in Frezza *et al*.[38]. Mitochondrial and cytosolic fractions were obtained and were blotted with a mouse monoclonal antibody anti-TOM20 (1:5000) ref. FL-145 (Santa Cruz Biotechnology Santa Cruz, USA) and a goat polyclonal antibody anti-Actin (1:2000) ref sc1616 (Santa Cruz Biotechnology Santa Cruz, USA). Cells were lysed in RIPA buffer, in the presence of phosphatase and protease inhibitors. Similar amounts of total protein were resolved by SDS-PAGE and transferred onto a nitrocellulose membrane (GE Healthcare, Little Chalfont, England). We have used as primary antibodies, the ones already referred in the immunohistochemistry section (all at a dilution of 1:1000). For protein detection, a horseradish peroxidase-conjugated secondary antibody (Santa Cruz Biotechnology, Santa Cruz, USA) and a luminescence system (Perkin-Elmer, Foster City, USA) were used. Membranes were re-probed with a mouse monoclonal antibody anti-TOM20 (1:5000) ref. FL-145 (Santa Cruz Biotechnology Santa Cruz, USA) for control of the protein loading. Protein expression was quantified using the Bio-Rad Quantity One 1-D Analysis software.

### Electron microscopy

Cells were prepared using a conventional electron microscopy fixation procedure and images were taken using a Technai 20 (FEI, Eindhoven, Netherlands). Differences in mitochondrion size between cell lines were calculated using the whole mitochondria surface area. A minimum of 15 mitochondria were measured per cell and at least 5 different cells per specimen were randomly used for the size measuring. All experiments were made in triplicate.

### Mitochondrial network imaging

To quantify structural mitochondrial network fragmentation, cells were grown in glass-bottom dishes, transfected with mitochondrial targeted yellow and red fluorescent protein, mtYFP and mtRFP respectively, and after 24h were fixed with ice-cold 3.7% formaldehyde for 30 minutes. Cells expressing mtRFP were imaged with the Zeiss 510 META confocal laser scanning microscope using a Plan-Apochromat 100/1.46NA objective with a 2x digital zoom (excitation at 561 nm, emission recorded above 575nm. For every cell line or condition at least 25 randomly selected cells were imaged and quantified according to its mitochondrial network. A 100x Plan-Apochromat (1.46NA) objective and 2x zoom were used to image single cells. Digital images were processed using ImageJ 1.47 (U.S. National Institutes of Health—NIH, Bethesda, MD, USA).

### Scratch-wound assay

Cells were grown to near 90% confluence in 6 well plates. Under aseptically conditions a thin "wound" was introduced by scratching with a 10μl pipette tip and detached cells were rinsed with PBS. Using a phase contrast set up and a 40x total magnification pictures were taken every 5 minutes for a total of 15 hours. Five different measurements per field were made using the pictures taken at 0, 3, 6, 9, 12 and 15 hours using ImageJ software. More detailed information on this methodology has been previously published[39]. Images were processed with ImageJ software.

### Migration/invasion assays

Transiently transfected TPC-1 and XTC.UC1 cells were resuspended in serum-free RPMI and with DMEM/F12, respectively, with 0.1% FBS and seeded in the upper chamber of a transwell 12-well plate (Corning Costar). The transwell plates were pre-coated with 5μg/ml fibronectin (F2006-1MG, Sigma Aldrich) and incubated at 37°C 5% CO_2_ for 4h. Plates were then rinsed with PBS1x at pH7.4 before adding the 100.000 cells per well. The lower chambers were filled with the respective medium supplemented with 10% FBS. After 12 hours of assay, the transwell membranes were excised, inverted and stained with DAPI on a glass slide. The nuclei visible at the facing-down side of the membrane were counted. A minimum of 5 different fields were counted for each slide and a triplicate was made for each experimental setting. Mdivi1 inhibitor was used at 50μM based on previous published data and in the absence of observable toxic or stress cell changes[40]. No differences were found between cells treated with Mdivi1 from time zero or pre-treated 1hour before the beginning of the assay.

### Statistical analysis

The statistical analysis of the IHC results was performed with STAT VIEW-J 5.0 (SAS Institute, Inc., Cary, NC). The relationship between the average expression level (score) of the immunohistochemical markers and the histopathology of the different cases were evaluated by ANOVA; results were considered statistically significant whenever p value < 0.05. When appropriate, multiple comparison corrections were performed using the post hoc Bonferroni/Dunn test and, upon program indication, comparisons were only considered significant whenever p value < 0.0018.

Every *in vitro* analysis was performed at least in triplicate and the mean thus obtained was used for independent Student’s t-test. All the data are expressed as mean ± SE, as indicated in the legends of the figures.

## Results

### Mfn2, Opa1, Drp1 and Fis1, are overexpressed in oncocytic cell tumors and Drp1 overexpression is associated with malignant oncocytic cell tumors

**Fig 1:**
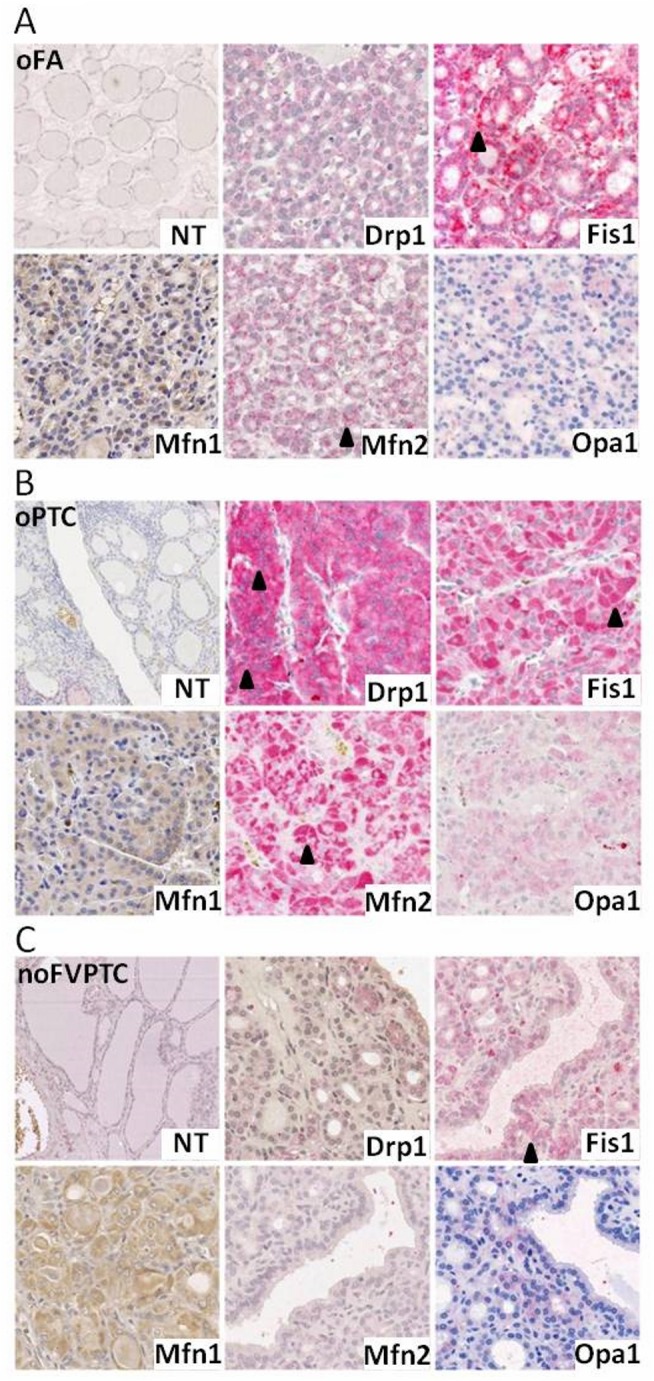
The expression levels’ analysis of mitochondria-shaping proteins shows an overall increase in oncocytic thyroid tumors when compared to their non-oncocytic counterparts. Representative micrographs showing immunostaining of Mfn1, Mfn2, Opa1, Drp1 and Fis1 antibodies in normal thyroid tissue (NT) and tumoral tissue of an oncocytic follicular adenoma—oFA) (A), an oncocytic papillary thyroid carcinoma—oPTC (B) and a non-oncocytic follicular variant of papillary thyroid carcinoma—noFVPTV (C) at a total magnification of 300x. Illustrative black arrowheads denote stained measured tumoral areas with an increased gradation from noFVPTV, to oFA and finally oFTC.” # Mfn1—Mitofusin 1, Mfn2—Mitofusin 2, Opa1—Optic atrophy 1, Drp1—Dynamin related protein 1, Fis1—Mitochondrial fission 1 protein.

**Fig 2:**
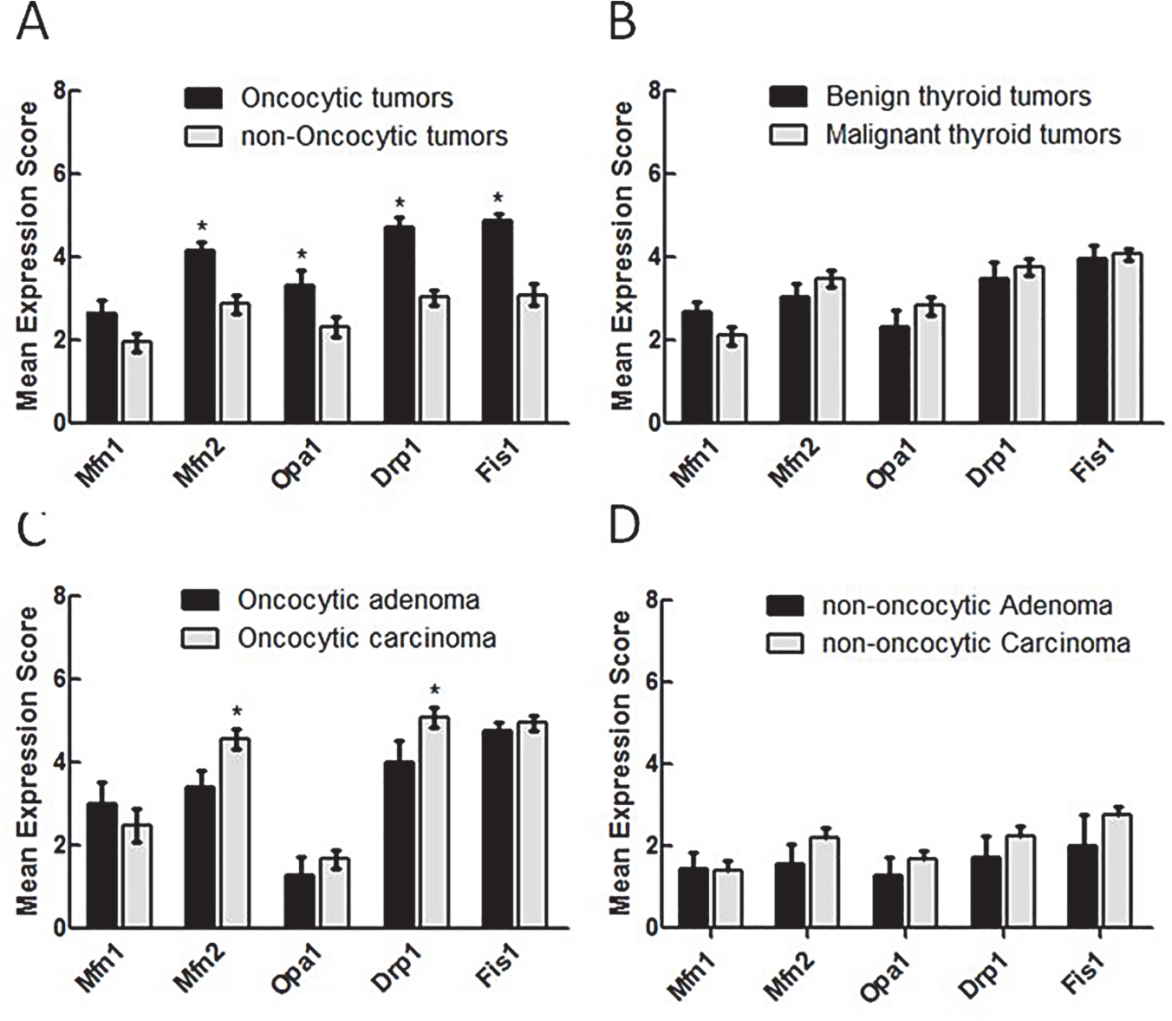
A significant increase on Drp1 expression is associated with malignant oncocytic thyroid tumors. The immunostaining of Mfn1, Mfn2, Opa1, Drp1 and Fis1 was evaluated, taking into account all thyroid tumors (A and B), the oncocytic thyroid tumors alone (C) or the non-oncocytic thyroid tumors alone (D). The mean antigen expression was calculated as described in Material and Methods. Results are shown as mean score ±SEM. * p<0.05 (unpaired t-test). # Mfn1—Mitofusin 1, Mfn2—Mitofusin 2, Opa1—Optic atrophy 1, Drp1—Dynamin related protein 1, Fis1—Mitochondrial fission 1 protein.

The so-called "mitochondria-shaping" proteins are key regulators of the mitochondria shape and of their dynamics. The mitochondrial network, indeed, is defined by the highly regulated balance between fusion and fission processes, depending on the cell’s physiological needs. Among several cellular processes and patho-physiological conditions, the mitochondria-shaping proteins also act on the homeostasis of the organelles size and number[4,28,29,32]. Once the oncocytic cell tumors differ from the non-ococytic ones by an abnormal accumulation of mitochondria with an altered morphology in the cell cytoplasm, we hypothesized that mitochondrial dynamics might have a role in the phenotypic and physiological oncocytic definition[11,12,13]. Thus, we have performed a histological analysis of the main "mitochondria-shaping" protein levels on several histological human thyroid tumor samples. Fig 1 shows representative immunostaining micrographs of the "mitochondria-shaping" proteins observed in different histological types of thyroid tumors, including 1 oncocytic follicular thyroid carcinoma (FTC), 6 non-oncocytic FTC, 8 oncocytic follicular variant of papillary thyroid carcinoma (FVPTC), 22 non-oncocytic FVPTC, 9 oncocytic conventional papillary thyroid carcinoma (cPTC), 23 non-oncocytic cPTC, 12 oncocytic follicular thyroid adenoma (FA) and 7 non-oncocytic FA. IHC micrographs representative of a benign oncocytic lesion (oFA), a malignant oncocytic lesion (oPTC) and a non oncocytic lesion (the noFVPTC), the histotypes of main interest in this work, are presented in Fig 1A, 1B and 1C. Since the expression of "mitochondria-shaping" proteins in the normal adjacent parenchyma was zero, or only detectable below the score 2, we omitted in the histograms the expression levels of such proteins in normal tissue. With the exception of Mfn1, we identified an increase in the expression levels of the other proteins studied, in the oncocytic cell tumors *vs*. the non-oncocytic ones and in all four histo-types analyzed (FVPTC, FTC, cPTC and FA) (Figs 1 and 2A).

### Mfn2 and the pro-fission Drp1 are significantly overexpressed in malignant oncocytomas

In a deeper analysis of the histological samples from human thyroid tumors, Mfn1 was the only mitochondria-shaping protein to show no significant difference in the expression levels, independently of the phenotype or tumoral types compared (Fig 2A–2D). An overall increase of the levels of the other proteins analyzed, independently of their pro-fusion or pro-fission role, were all significantly higher in the oncocytic tumors compared to in the non-oncocytic ones (4.2±0.2 *vs*. 2.9±0.2, p<0.0001 for Mfn2; 3.3±0.3 *vs*. 2.3±0.2, p = 0.01 for Opa1; 4.7±0.2 *vs*. 3.0±0.2, p<0.0001 for Drp1; 4.9±0.1 *vs*. 3.5±0.2, p<0.0001 for Fis1), as shown in the IHC quantification of Fig 2A (Fig 2A). In contrast, there was no appreciable difference for any of these proteins, when we compared malignant *vs*. benign tumors, without distinguishing and grouping the samples into oncocytic or non-oncocytic (3.5±0.2 *vs*. 3.0±0.3, p = 0.31 for Mfn2; 2.8±0.2 *vs*. 2.3±0.4, p = 0.28 for Opa1; 3.8±0.2 *vs*. 3.5±0.4, p = 0.48 for Drp1; 4.1±0.1 *vs*. 3.9±0.34, p = 0.71 for Fis1) (Fig 2B). Therefore, it seems that the alteration of the machinery responsible for mitochondrial morphology is strictly related to the oncocytic phenotype, rather than to the overall tumor aggressiveness. Furthermore, within the non-oncocytic tumors no differences in the expression levels of mitochondrial dynamics proteins were found between benign and malignant tumors (Fig 2D). However, if we consider the degree of severity of the tumor, only within the oncocytic phenotype, interestingly we observe that Mfn2 and Drp1 are significantly more abundant in carcinomas compared to the benign counterparts’ adenomas (4.5±0.2 *vs*. 3.4±0.4, p = 0.012 for Mfn2 and 5.1±0.2 *vs*. 4.0±0.5, p = 0.038 for Drp1 (Fig 2C). Of note, these differences are not related to differences in mitochondrial number (S1 Fig). This suggests that within oncocytic cell tumors, the mitochondrial dynamics assume a role in the definition of tumor malignancy and aggressiveness.

### Drp1 actively accumulates in the mitochondria and unbalance mitochondrial network towards fission

In order to clarify and investigate in depth the role of mitochondrial dynamics alteration on oncocytic cell tumors, we switched to *in vitro* experiments on two thyroid cancer cell lines: XTC.UC1, which is the only oncocytic cell line available, with carcinoma derivation, and TPC1, a non-oncocytic thyroid cell line, used for comparative purposes. First, we evaluated in the two cell lines the expression level of the same fusion/fission proteins studied in the human tumor samples.

**Fig 3:**
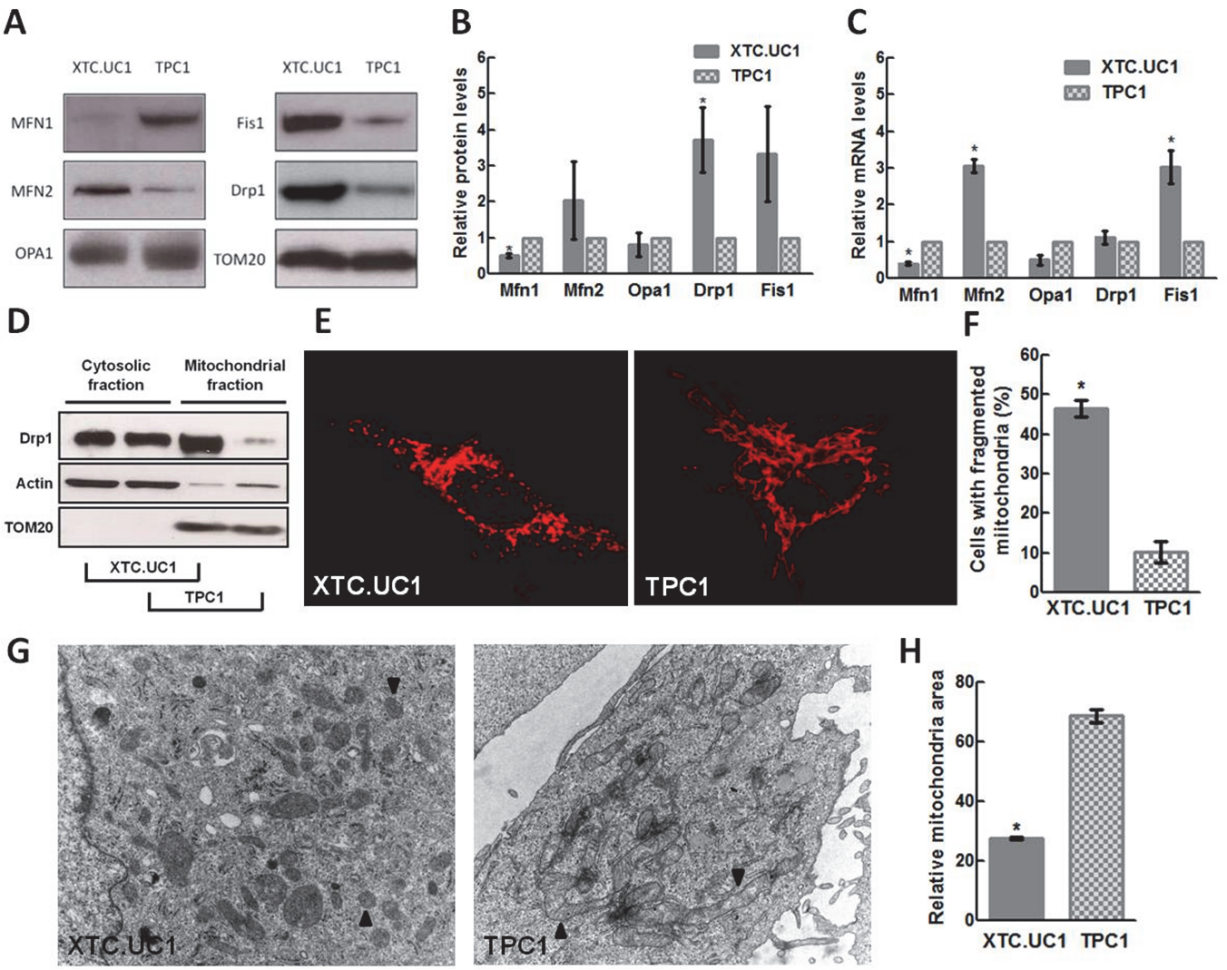
*In vitro* characterization of mitochondria in oncocytic XTC.UC1 and non-oncocytic TPC1 cell lines. Mitochondrial fission proteins are overexpressed in oncocytic cancer cells and Drp1 actively accumulates on mitochondria. (A and B) Representative western blot analysis of Mfn1, Mfn2, Opa1, Drp1 and Fis1 expression levels in protein extracts of XTC.UC1 and TPC1 show an overexpression of mitochondrial fission proteins and a downregulation of pro-fusion Mfn1. The levels of Mfn1, Mfn2, Opa1, Drp1 and Fis1 were quantified and normalized by the levels of control protein (TOM-20). Results are shown as mean expression value ± SEM of at least three independent experiments.* p<0.05 (unpaired t-test). (C) Drp1 mRNA levels were similar in XTC.UC1 and TPC1 cell lines. mRNA levels of Drp1, Fis1, Mfn2, Mfn1 and Opa1 were accessed by qPCR in XTC.UC1 and TPC1 cell lines. Results are expressed as relative expression for each individual gene normalized to the respective control protein (Actin) between cell lines. Results are shown as mean expression value of at least three independent experiments. Error bars represent SEM. * p<0.05 (unpaired t-test). (D) Drp1 actively accumulates at mitochondria in the XTC.UC1 cell line. Representative western blot analysis of equal protein extracts from the heavy membrane fraction and cytosolic fraction is shown in both XTC.UC1 and TPC1 cell lines. (E and F)- Mitochondrial network is fragmented in almost half of the cellular population of XTC.UC1. Representative confocal images of XTC.UC1 and TPC1 cell lines transfected with mitochondrial targeted RFP (mtRFP) showing mitochondrial fragmentation in XTC.UC1 and TPC1 at basal conditions. XTC.UC1 cell line shows a 4-fold increase in the number of cells with fragmented mitochondrial network in comparison to TPC-1. The number of cells with fragmented mitochondria was quantified by counting at least 30 cells per field. Results are shown as mean expression value ± SEM of at least three independent experiments.* p<0.05. # Mfn1—Mitofusin 1, Mfn2—Mitofusin 2, Opa1—Optic atrophy 1, Drp1—Dynamin related protein 1, Fis1—Mitochondrial fission 1 protein and TOM20—translocase of outer mitochondrial membranes 20 kDa. XTC.UC1—oncocytic cell follicular carcinoma, TPC1—Papillary thyroid carcinoma-derived cell line. (G and H) Electron microscopy showed significantly smaller mitochondria in XTC.UC1. Representative electron microscopy images of XTC.UC1 and TPC1 cells are shown, using a total magnification of 5800x. Black arrowheads denote differences in size. The mitochondrial size is less than 2.5 times lower in XTC.UC1 than in TPC1. Mitochondrial area was estimated by measuring the outer contour of a minimum of 15 mitochondria per cell. At least 5 different cells per specimen were randomly used for the size estimation. Results are shown as mean expression value ± SEM of at least three independent experiments.* p<0.05. **#** XTC.UC1—oncocytic cell follicular carcinoma, TPC1—Papillary thyroid carcinoma-derived cell line.

By western blot analysis, we detected, in the oncocytic cell line, the same trend of expression pattern of "mitochondria-shaping" proteins, as we observed in oncocytic thyroid tumors, i.e. an increase in the mitochondrial fission proteins Drp1 (p<0.05) and Fis1 (although not statistically significant) in XTC.UC1 cell line in comparison with TPC1 cell line (Fig 3A and 3B). Regarding the fusion proteins, we observed a statistically significant downregulation of Mfn1 (p<0.05) in XTC.UC1, a non-statistically significant increased expression level of Mfn2 and no differences in Opa1 (Fig 3A and 3B). Interestingly, at variance with the significant increase of Drp1 protein expression in XTC.UC1 when comparing with TPC1 cell line, Drp1 mRNA levels were similar in both cell lines (Fig 3C).

We decided to further investigate Drp1 in thyroid tumors’ oncocytic phenotype because we observed a statistically significant increase of Drp1 protein, both in oncocytic tumors and in the oncocytic cell line, and because of the known involvement of this protein in breast cancer malignancy[31].

Given that Drp1 is a cytosolic protein that translocates to mitochondria to be functionally active and take part in mitochondrial division, we wondered if Drp1 was actively recruited to the mitochondrial membrane or if it accumulated passively at the cytosol. To clarify this issue, we performed a sub-cellular fractioning in both cell lines and, we assessed the levels of Drp1 bound to the organelles on the heavy membrane fraction enriched on mitochondria. The levels of this protein were around 8 times higher in XTC.UC1 than in TPC1 (p<0.05) in the mitochondria-rich fraction, much higher than that detected in the total cytosolic protein lysates (Fig 3D) and in cytoplasm; this indicated a substantial accumulation, and thus activation, of the pro-fission protein at mitochondria. Indeed, as expected at this point, confocal microscopy analysis on single cells, with a fluorescent protein specifically targeted to mitochondria (mtRFP) overexpressed in order to visualize the organelles, showed that mitochondria of the oncocytic cell line XTC.UC1 are much more fragmented than the non-oncocytic ones (46,5% *vs*. 10,2%) (Fig 3E and 3F).

By electron microscopy, we were able to confirm this morphological imbalance toward fission observed by WB and qPCR in the oncocytic XTC.UC1 cells as denoted by the black arrowheads (about 2.5 times smaller mitochondria in XTC.UC1 than in TPC1, p<0.05) in Fig 3G and 3H.

### Oncocytic cancer cell line faster migration is sensitive to genetic and pharmacological blockage of Drp1

**Fig 4:**
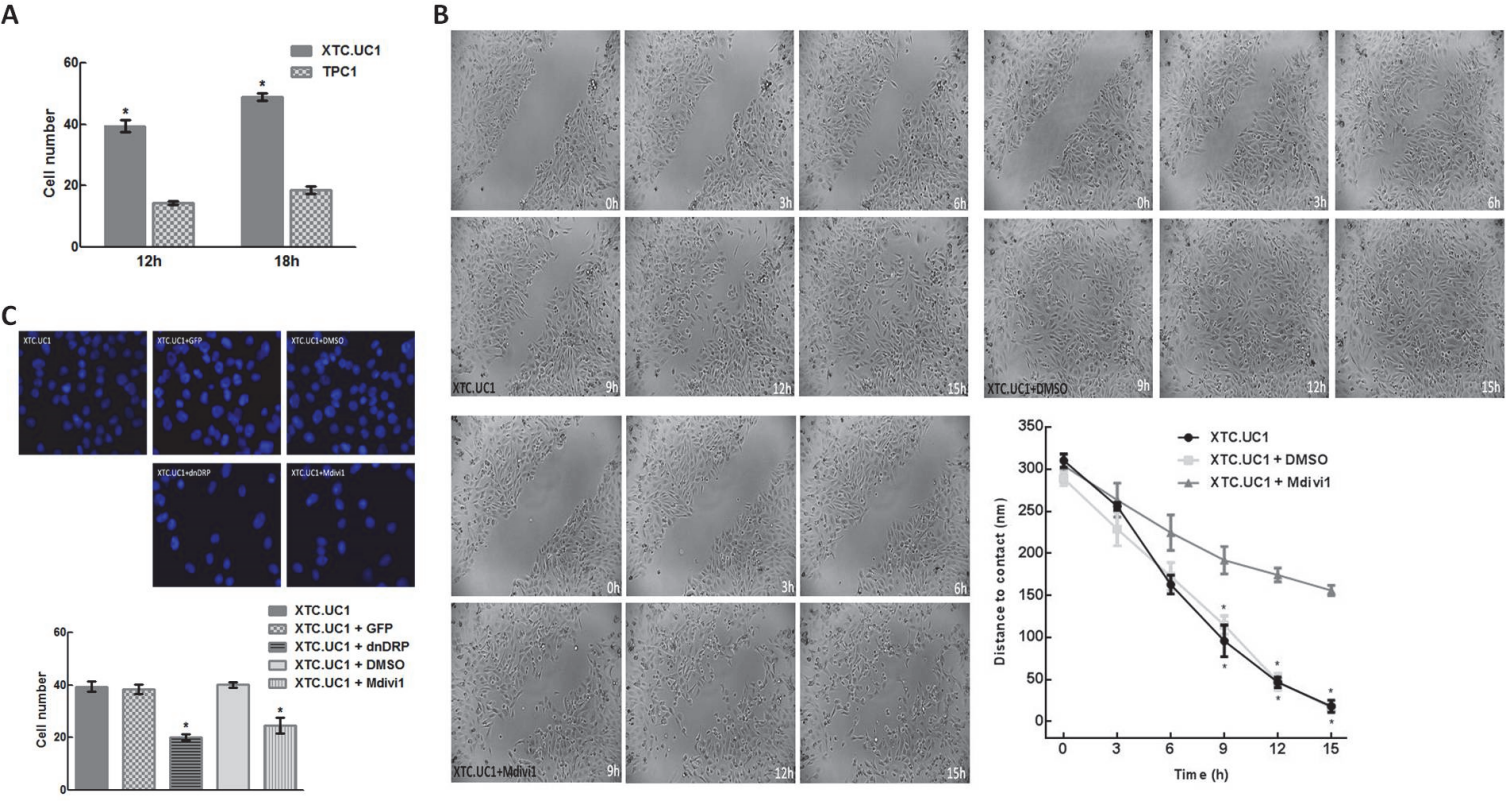
Pharmacological and genetic blockage of Drp1 prevent oncocytic cell migration/invasion. (A) XTC.UC1 cells show higher basal migration ability on a transwell assay. A minimum of 5 different fields were counted for each slide and triplicates were made for each experimental setting. (B) Pharmacological blockage of Drp1 significantly restrains oncocytic cell (XTC.UC1) migration in a wound-healing assay. Representative frames acquired at the indicated time points (every 1 hour) during a 15h wound-healing assay performed in XTC.UC1 untreated, XTC.UC1+DMSO and XTC.UC1+Mdivi1 (a specific Drp1 inhibitor) cells are here shown. Results are shown as mean expression value ± SEM of at least three independent experiments. * denotes p < 0.05 in a paired sample t-test. (C) Pharmacological or genetically blocking Drp1 results in diminished XTC.UC1 cell migration/invasion ability in a transwell assay. Representative images taken at the end of a 12hour time point transwell assay showing a decrease in the migration/invasion ability of cells treated both with a genetic (XTC.UC1+dnDRP) and pharmacological (XTC.UC1+Mdivi1) inhibition of Drp1. Conditions: XTC.UC1 untreated, GFP-transfected XTC.UC1 (as a transfection control), GFP+dnDRP co-transfected XTC.UC1, XTC.UC1+DMSO (as a pharmacological blockage control) and XTC.UC1+Mdivi1 (a specific Drp1 inhibitor). Results are shown as mean expression value ± SEM of at least three independent experiments. * p < 0.05 in a paired sample t-test. **#** XTC.UC1—oncocytic cell follicular carcinoma; GFP—green fluorescent protein; dnDRP—dominant negative form of dynamin related protein 1 (Drp1) Drp1-K38A; DMSO—Dimethyl sulfoxide; Mdivi1—selective cell-permeable inhibitor of mitochondrial division Drp1.

Since it has been previously shown that mitochondrial dynamics, namely Drp1, play a crucial role in orchestrating lymphocyte chemotaxis and migration of metastatic breast cancer, we set out to establish if the Drp1 pro-fission protein overexpression correlated with a higher capability of migration in the oncocytic XTC.UC1 cell line[30,31]. Accordingly we decided to perform a scratch-wound assay. This is a commonly used method to measure basic cell migration parameters, by following, in timelapse, how fast confluent cells fill in a wound manually scratched in a culture plate. We observed through this assay that XTC.UC1 cells have, indeed, higher motility and need less time to fill in the wound space than TPC1 cells (S2 Fig). We then confirmed this result by transwell assay, a test frequently used to study cell migratory response and invasion ability toward a specific chemical gradient created by a fibronectin-coated artificial filter. In this case, either at a 12 or 18 hours interval, we obtained more than double the number of cells able to transpose the fibronectin coated transwell membranes in XTC.UC1 than in TPC1 (Fig 4A).

To evaluate if the aforementioned higher ability to move and migrate could be attributed to the overexpression of the pro-fission Drp1 found in XTC.UC1, we interfered, both pharmacologically and genetically, with this cell line’s fission machinery by blocking the Drp1 activity. First, we performed a scratch-wound assay in the presence of Mdivi1, a specific Drp1 inhibitor blocking its assembly and translocation to mitochondria[40]. The pharmacological block of Drp1 did indeed reduce the cell motility by almost 50% in comparison to untreated cells. This was accompanied by a reduction in the number of cells with fragmented mitochondria in favor of an increase of cells with an interconnected mitochondrial network (Fig 4B and S3 Fig). A transwell assay performed under the same conditions confirmed our observations on the migration reduction (Fig 4C). To reinforce this interesting result, we also assessed the migration/invasion competence of XTC.UC1 cells by genetically interfering with the fission machinery, specifically targeting Drp1 activity. We performed a co-transfection with a mitochondrial targeted fluorescent protein mtGFP and a dominant negative form of Drp1 (dnDrp1) Drp1-K38A, a form of Drp1 with a mutation in its GTPase catalytic domain that blocks its activity. We then analyzed cell migration by transwell assay. Again, we observed the same trend of cell chemotaxis inhibition (Fig 4C), thus indicating the dependence on Drp1 of the migratory process. This set of experiments unraveled a role for Drp1 and mitochondrial fragmentation on modulating the migratory capability in oncocytic thyroid cancer cells.

## Discussion

Our study on a subset of human thyroid oncocytic tumors sheds light on a pivotal role of mitochondrial dynamics in determining this specific cancer phenotype and, at least partially, its malignant features. Our data highlight an alteration in the mitochondrial network regulation, particularly relevant in oncocytic tumors, and culminating in a weakened fusion and enhanced fission of the organelles.

The fact that both the pro-fusion proteins Mfn2 and Opa1 were more expressed in oncocytic thyroid tumors than in their non-oncocytic counterparts may be related to other known cellular of these proteins: Mfn2-dependent ER-mitochondria tethering and Opa1 anti-apoptotic role in maintaining a proper cristae structure[27,28]. We can speculate that Mfn2 could regulate calcium signaling and ER stress-induced cell death, thus delaying apoptosis. Alternatively, it may facilitate ATP generation in the thyroid model as in the case of cardiac and skeletal muscle setting[41,42]. Although Opa1 was overexpressed in oncocytic thyroid tumor samples, no significant difference was found in cell lines. This apparent discrepancy may reflect tumor cells’ adaptive response to specific tumor microenvironmental conditions that might not be related to Opa1’s fusion activity[43].

Moreover, the decrease in Mfn1 levels in the oncocytic cell line, could have the functional meaning of delaying the oxidative stress-induced loss of mitochondrial membrane in such a highly-rich ROS environment as in the case of mitochondria-rich tumors (as observed in cardiomyocytes[44]), although this might not justify *per se* the observed increase in the mitochondrial network fragmentation. Such a finding is most likely related to alterations in the fission machinery. Indeed, both *ex vivo* and *in vitro* data also showed an overexpression of the fission proteins Drp1 and Fis1 in the oncocytic thyroid tumors histotype, favoring fission over fusion. Thus, the high amount of small mitochondria in these cells most probably results from a combination of both increased fission and decreased fusion processes. Fis1 is permanently anchored to mitochondria and, besides its role in fission, “behaves” like a mitochondrial structural protein. It therefore makes sense that mitochondrion-rich cells present an increase in Fis1 expression.

Regarding Drp1, we found that the mean expression levels in oncocytic thyroid tumors are higher than in non-oncocytic ones. No statistically significant differences between benign and malignant thyroid tumors were observed in the expression levels of Drp1; however, when we considered in our analysis only the oncocytic tumors, Drp1 expression levels were higher in carcinomas than in adenomas. This finding suggests that Drp1 may play a role in malignant transformation within the oncocytic thyroid tumor setting. Additionally, in regard to the non-oncocytic tumors, no differences in the expression levels of any protein were found between benign and malignant thyroid tumors. We believe that when we consider all thyroid tumors histotypes, these differences might become diluted; a possible correlation between mitochondrial dynamics proteins expression levels might thereby become lost, thus reinforcing the importance of these proteins in the oncocytic group.

In our *in vitro* results it is interesting to note that, although no differences were found in Drp1 mRNA levels when comparing the two thyroid cell lines, by western blot analysis the oncocytic XTC.UC1 presented significantly higher expression Drp1 levels than did the non-oncocytic TPC1. Drp1 exists as a cytosolic protein which actively needs to translocate to the mitochondrial outer membrane to promote organelle division. Several forms of post-translational regulation of Drp1 have been reported; it is possible that in oncocytic tumors, Drp1 could be stabilized, namely by Ser-616 phosphorylation or Ser-637 dephosphorylation, and translocated to mitochondria, thus promoting an unbalanced organelle division as previously described[45,46,47,48]. We saw that the oncocytic cell line displays mitochondria which are particularly small and often with an aberrant shape. We also observed a significant co-localization of Drp1 with the mitochondrial membrane upon heavy membrane fraction analysis in the same model. We then logically went on to investigate on these cells’ migratory capability.

By scratch-wound and transwell assay we observed that oncocytic XTC.UC1 cells have higher migration/invasion capacity. XTC.UC1 being a cell line derived from a metastatic tumor, it makes sense that it has a higher motility and migration ability than TPC1. These observations are not biased by differences in cell proliferation rates since the proliferation rate is higher in TPC1 than in XTC.UC1 cell line[49].

Given that we observed Drp1 overexpression in the oncocytic cell line as well in the oncocytic tumors, where it associates with malignancy, we hypothesize that the Drp1 protein may have a role in the observed higher migration/invasion abilities of the XTC.UC1 cells. Our hypothesis was supported by the observation that the inhibition of Drp1 function, both pharmacologically and genetically, leads to a decrease in the migration/invasion capacity of the oncocytic XTC.UC1 cells. Our results match previously published data showing that mitochondrial dynamics regulates lymphocyte and breast cancer cell migration[30,31]. In both these experimental models, either inhibiting the fission process by Drp1 downregulation or promoting a hiperfused mitochondrial network phenotype, by overexpressing Mfn1, led to limited cell migration or invasion. In our case also, a block of Drp1 was sufficient to reduce the oncocytic cell migration.

Our hypothesis would be better verified if we could use other cell lines, namely a cell line derived from an oncocytic adenoma and one from a non-metastatic primary oncocytic carcinoma. However these cell lines do not exist. Alternatively, we cannot rely on primary cultures of oncocytic and non-oncocytic, benign or malignant thyroid tumors, since unfortunately the culture of primary thyroid cells has been very difficult to obtain and do not last in the laboratory long enough for us to perform the studies. Those cell lines, if available, would be valuable tools for understanding not only thyroid tumors’ oncocytic transformation, but also their malignant transformation.

To sum up, our data show that the mitochondrial fission proteins, Drp1 and Fis1, are overexpressed in thyroid oncocytic cell tumors. This suggests that the mitochondrial fission process upregulation might contribute to mitochondrial accumulation in tumor cells and thus be responsible, at least partially, for the oncocytic cell phenotype. Furthermore, we found that mitochondrial fission protein expression, in particular Drp1 overexpression, correlates with oncocytic thyroid tumor malignancy *ex vivo* and also with a higher migration/invasion ability of the XTC.UC1 cell line, an oncocytic cell line derived from a metastatic malignant thyroid tumor.

Further studies on the mitochondrial dynamics are needed to better dissect some of the cellular dysfunctions observed in tumor cells in general and in oncocytic tumor cells in particular.

## Supporting Information

S1 Fig Representative micrographs showing that immunostaining of the mitochondrial control protein SDHA—Drp1 expression levels is not dependent on the mitochondrial content/number. (TIF)

S2 Fig XTC. UC1 cells have higher motility and need less time to fill in the wound.
(TIF)

S3 Fig Mdivi1 treatment blocks Drp1 activity and promotes a decrease in the percentage of XTC.UC1 cells with fragmented mitochondria. (TIF)
