# Supplementary figures and images for: Mitochondrial Dynamics Protein Drp1 Is Overexpressed in Oncocytic Thyroid Tumors and Regulates Cancer Cell Migration
Source: PLoS One. 2015 Mar 30;10(3):e0122308. doi: 10.1371/journal.pone.0122308 (PMC4379140; doi:10.1371/journal.pone.0122308)

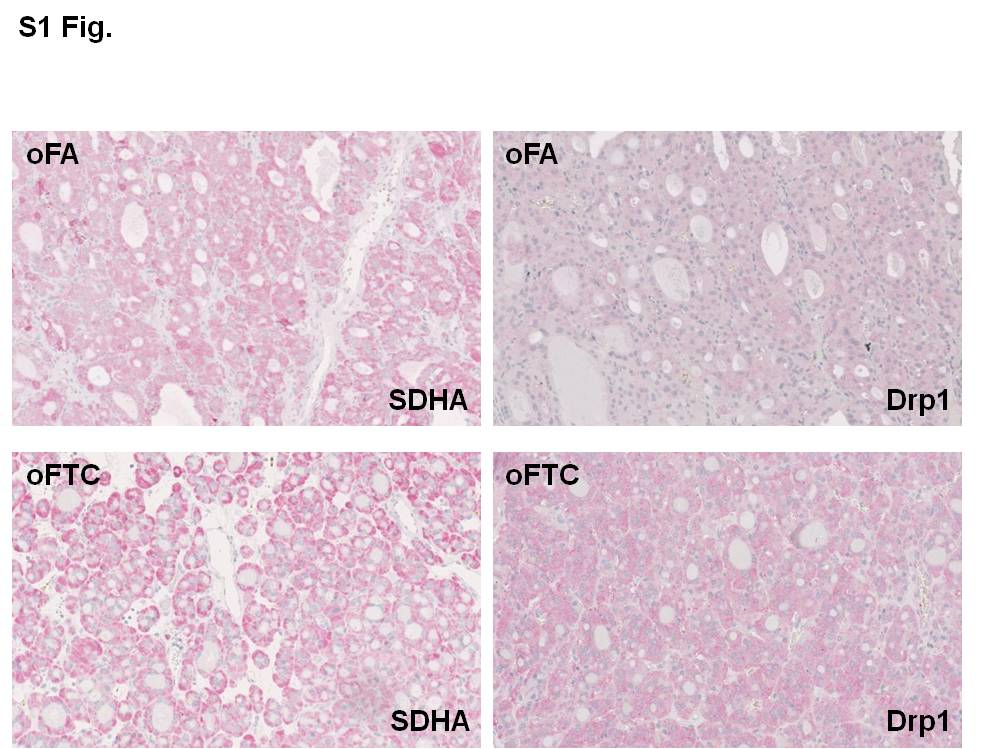

Supplement: S1 Fig — (TIF) [file pone.0122308.s001.tif]

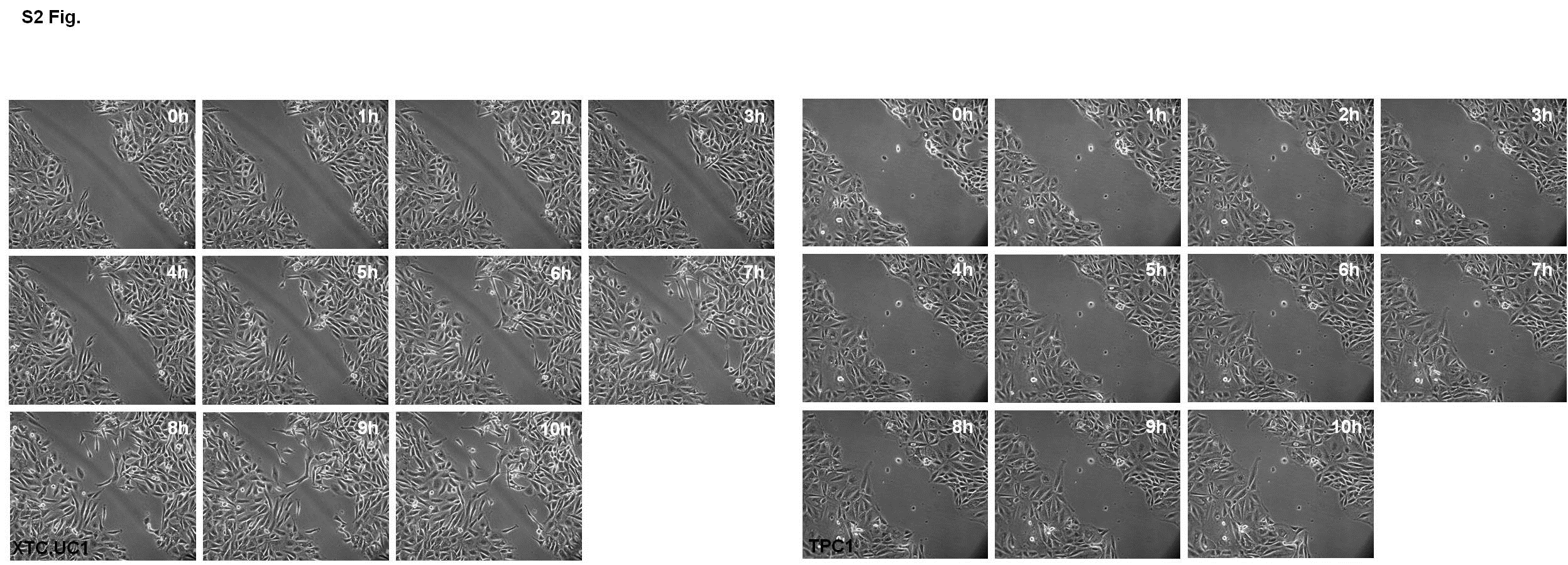

Supplement: S2 Fig — UC1 cells have higher motility and need less time to fill in the wound. (TIF) [file pone.0122308.s002.tif]

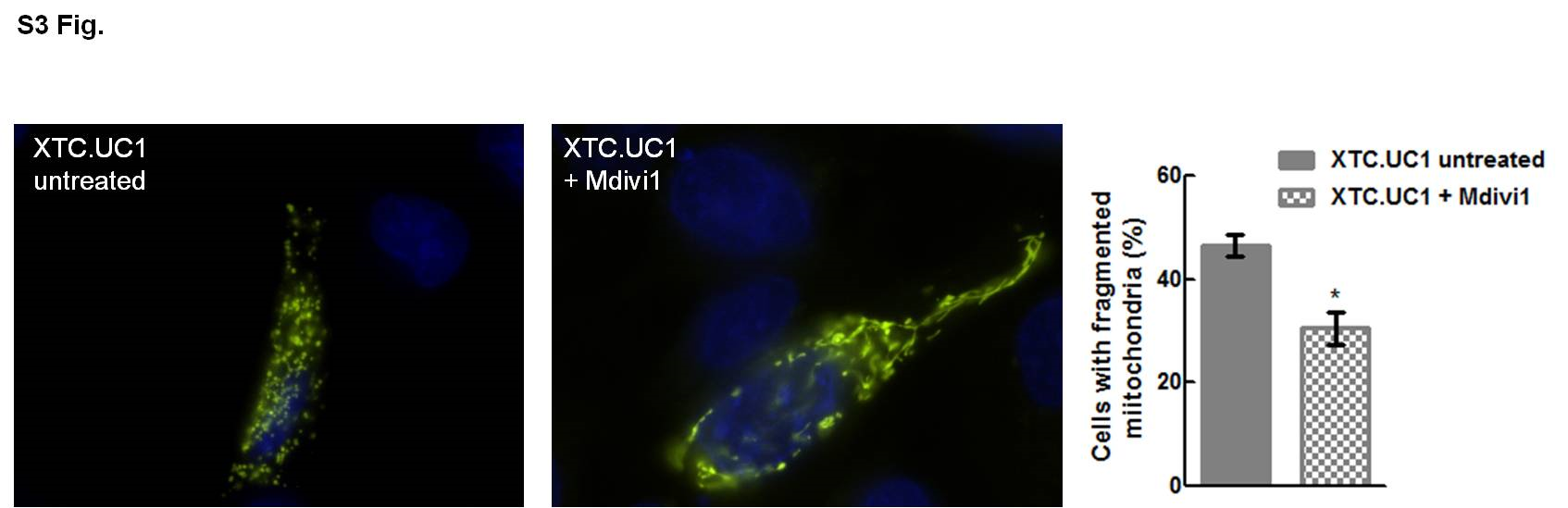

Supplement: S3 Fig — (TIF) [file pone.0122308.s003.tif]
